# Pregnancy outcomes in freeze-all versus fresh embryo transfer cycles of women with adenomyosis and endometriosis: a systemic review and meta-analysis

**DOI:** 10.3389/fendo.2025.1507252

**Published:** 2025-05-14

**Authors:** Yixian Han, Chang Liu, Dong Liu, Lukanxuan Wu, Wei Huang

**Affiliations:** ^1^ Department of Obstetrics and Gynecology, West China Second University Hospital of Sichuan University, Chengdu, Sichuan, China; ^2^ Key Laboratory of Birth Defects and Related Diseases of Women and Children (Sichuan University), Ministry of Education, West China Second University Hospital of Sichuan University, Chengdu, Sichuan, China; ^3^ National Health Commission (NHC) Key Laboratory of Chronobiology, Sichuan University, Chengdu, Sichuan, China

**Keywords:** endometriosis, adenomyosis, assist reproductive technology, freeze-all, embryo transfer, pregnancy outcomes

## Abstract

**Background:**

Endometriosis (EMS) and adenomyosis have adverse effects on women’s fertility. *In vitro* fertilization (IVF) and intracytoplasmic sperm injection (ICSI) are effective treatments for these diseases. Research has shown that different embryo transfer strategies in IVF/ICSI can influence gestational outcomes. This systematic review and meta-analysis aimed to evaluate the impact of freeze-all embryo transfer (FET) versus fresh embryo transfer (ET) strategies in IVF/ICSI cycles for infertile women with EMS and adenomyosis.

**Method:**

A comprehensive search was conducted across PubMed, EMBASE, MEDLINE, Web of Science, Google Scholar, and Chinese databases to identify studies examining different embryo transfer strategies in IVF/ICSI cycles among patients with EMS and adenomyosis. The outcomes analyzed included rates of implantation, clinical pregnancy, miscarriage, and live birth. Odds ratios (ORs) with 95% confidence intervals (CIs) were calculated using random-effects or fixed-effects models.

**Results:**

In patients with EMS, the results demonstrated that the FET strategy yielded higher clinical pregnancy (OR: 1.25; 95% CI: 1.11, 1.40), live birth rates (OR: 1.31; 95% CI: 1.15, 1.49), and implantation rates (OR: 1.27; 95% CI: 1.05, 1.54) compared to the fresh ET strategy. The miscarriage rate (OR: 0.89; 95% CI: 0.52, 1.52) and the ectopic pregnancy rate (OR: 0.51; 95% CI: 0.24, 1.07) were comparable between groups. For the group of women with adenomyosis, the IVF/ICSI outcomes were comparable between the FET and fresh ET strategies.

**Conclusion:**

In IVF/ICSI, the FET strategy has been associated with more favorable reproductive outcomes compared to the fresh ET strategy in women with EMS. Whereas in women with adenomyosis, pregnancy outcomes were comparable between the FET and fresh ET groups.

**Systematic Review Registration:**

https://www.crd.york.ac.uk/PROSPERO/view/CRD42024563268, identifier CRD42024563268.

## Introduction

Endometriosis (EMS) and adenomyosis are interrelated chronic diseases, both originating from ectopically located intracavitary endometrium. EMS is characterized by the presence of endometrial stroma and glands outside the uterine cavity, while adenomyosis is defined by the infiltration of endometrial tissue within the myometrium (1). Women with EMS and adenomyosis often suffer from subfertility and infertility (2). Up to 35–50% of infertile women are affected by EMS, while the prevalence of adenomyosis in infertile women is reported to be approximately 7.5-24.4% (3). The pathological processes may involve inflammation and fibrosis, immune modulation, altered steroid hormone metabolism, increased oxidative stress, and intrauterine abnormalities. These factors potentially interfere with folliculogenesis, sperm function, embryo transport, and endometrial receptivity (4, 5).

*In vitro* fertilization (IVF) or intracytoplasmic sperm injection (ICSI) and embryo transfer is a valid option for infertile women with EMS and adenomyosis (6, 7). The current clinical policy of transferring includes both fresh embryo transfer (ET) and freeze-all embryo transfer (FET). The FET strategy, initially designed to mitigate ovarian hyper-stimulation syndrome, facilitates embryo cryopreservation for subsequent suitable cycles. In IVF/ICSI, the key factors to conception are embryo quality, embryo-endometrium interaction, and endometrial receptivity (8). Recently, heightened attention has been drawn to the elevated sex steroid levels resulting from hyper-stimulation during controlled ovarian hyperstimulation (COS). It may exacerbate endometrial receptivity issues, reducing the likelihood of successful conception. As a results, there has been a proposal that FET approach can separate the COS process from the embryo transfer, thereby serving to circumvent the potential adverse impacts of COS on the endometrium (9). One randomized Controlled Trials (RCTs) involving patients with ovulatory women demonstrated favorable outcomes in either pregnancy outcomes or the incidence of ovarian hyper-stimulation syndrome for the FET group (10). With the advancement of vitrification techniques, FET approach has been applied to patients with EMS and adenomyosis. Bourdon et al. investigated 270 infertile women with EMS undergoing IVF/ICSI. Their results indicated that the FET strategy yielded higher cumulative clinical pregnancy and live birth rates compared to the fresh ET strategy (11). Similarly, a retrospective cohort analysis found that the FET strategy in women with adenomyosis was associated with significantly higher odds of live birth compared to fresh ET (12). However, other studies reported comparable pregnancy outcomes among women with EMS or adenomyosis, regardless of whether FET or fresh ET was used (13–15). Additionally, Roque et al. conducted a systematic review and meta-analysis, demonstrating that FET significantly improved live birth rates compared to fresh ET, particularly in hyper-responders and preimplantation genetic testing for aneuploidy cycles (16). However, the lack of distinction between study populations limited its clinical applicability. To evaluate embryo transfer strategies in infertile patients with endometriosis and adenomyosis, we conducted a systematic review and meta-analysis.

## Materials and methods

### Protocol and registration

This systematic review and meta-analysis were conducted following the Preferred Reporting Items for Systematic Reviews and Meta-Analyses (PRISMA) guidelines (17). The protocol of this meta-analysis was registered in PROSPERO (CRD42024563268).

### Study design

We conducted a systematic review and meta-analysis compared the pregnancy outcomes after different embryo transfer strategies in patients with EMS and adenomyosis. In all cases, EMS and adenomyosis were diagnosed by biopsy through surgery or medical imaging evidence like transvaginal ultrasound (TVUS) or magnetic resonance imaging (18). In TVUS, ovarian endometrioma appeared as a persistent unilocular or multilocular cyst with homogeneous low-level echogenicity of the cyst fluid and absent or moderate cyst wall vascularization. Deeply infiltrating endometriosis in the TVUS appeared as thick tissue blocks, nodular formations, or irregularly shaped hypoechoic, commonly affecting the uterosacral ligament, pouch of Douglas, and/or vagina. In addition, adenomyosis showed myometrial cystic areas, hyperechoic islands, linear striations, and buds or irregular/infiltrated endometrial-myometrial junction zones (19–21).

First, we conducted a comparative analysis across the entire study population, including individuals with EMS or adenomyosis. Subsequently, we performed a subgroup analysis based on the included studies. One analysis comprised nine studies focusing on EMS, while the other included two studies on adenomyosis. We separately summarized the gestational outcomes for patients with EMS or adenomyosis.

### Search strategy

A systematic search for relevant papers was carried out in PubMed, EMBASE, MEDLINE, Web of Science, Google Scholar, China National Knowledge Infrastructure, Wanfang Data Knowledge Service Platform, and China Biomedical Literature Database. English search keywords included “endometriosis”, “adenomyosis”, “*in vitro* fertilization/IVF-ET”, “Intracytoplasmic sperm injection/ICSI”, “freeze-all/frozen embryo transfer”, “Fresh Embryo Transfer”, and “pregnancy outcomes”, as showed in support information. No restrictions were placed on the language or publication date of the studies. Additionally, we examined the reference lists of the eligible studies and review articles to identify any additional relevant articles.

### Inclusion and exclusion criteria

Studies were included in this meta-analysis if they matched the following inclusion criteria (1): subjects of study were women diagnosed with either EMS or adenomyosis. (2) study focused on the pregnancy outcomes of different embryo transfer types in IVF/ICSI cycles. The exposure group consisted of women undergoing FET, while the control group comprised those undergoing fresh ET. (3) the outcomes include at least one of the following: implantation rate, miscarriage rate, clinical pregnancy rate, ectopic pregnancy rate, or live births rate. (4) randomized controlled trials (RCTs) or cohort studies. exclusion criteria: (1) study involved with donor or recipient oocyte treatments. (2) Review articles, abstracts, letters, conference papers, and case reports were excluded. (3) Necessary data was not available. (4) studies included animal experiments.

### Study selection

All titles and abstracts were independently reviewed by two investigators (Yixian Han and Lukanxuan Wu). Studies that potentially met the inclusion criteria were further assessed through full-text review. Any discrepancies were resolved by consulting a third author(Chang Liu). The kappa statistic was used to evaluate inter-examiner agreement in study selection (22). Both researchers manually extracted data from the included studies using specially designed data collection forms. The following data were collected: lead author, publication year, country, study period, study design, intervention, age, body mass index, duration of infertility, diagnosis, EMS phenotype or stage, stimulation protocol, FET protocol, oocyte retrieved, and pregnancy outcomes.

### Risk of bias assessment

The quality of the included studies was evaluated using the Newcastle Ottawa scale (NOS) for assessing observational studies based on selection, comparability, and outcome domains. The total score of this scale was 9 points (0–3: poor quality, 4–6: fair quality,7–9: good quality) (23).

### Outcome measure

The primary outcomes were clinical pregnancy and live birth rate, and the secondary outcomes were miscarriage, implantation rate, and ectopic pregnancy rate. The implantation rate was defined as the proportion of transferred embryos that were successfully implanted. Clinical pregnancy was confirmed by the presence of at least one gestational sac on ultrasound, including ectopic pregnancy. Ectopic pregnancy was defined as the detection of a gestational sac outside the uterine cavity using TVUS, or clinically diagnosed when no gestational sac is observed within the uterine cavity but serum hCG levels continue to rise. A miscarriage was defined as the loss of gestation before 28 weeks of pregnancy. Live birth was described as a live birth event after 28 weeks.

### Statistical analysis

Statistical analysis was performed with Review Manager 5.4 software. All the outcomes in our analysis were binary. Dichotomous data was analyzed using the Mantel–Hansel odds ratio and the CIs between groups. The I² statistic and Cochran’s Q-statistic were used to assess methodologic and clinical heterogeneity across studies. High heterogeneity was defined as I² ≥ 50% or p < 0.10, in which case a random-effects model was applied; otherwise, a fixed-effects model was used. Sensitivity analysis and subgroup analysis were conducted when I² or Cochran’s Q-statistic detected significant heterogeneity. Funnel plot analysis was used to assess the potential publication bias. All statistical tests were two-sided, and *p* values < 0.05 were considered statistically significant. The GRADE approach was employed to evaluate the quality of each outcome (24). This assessment considered factors such as risk of bias, inconsistency, indirectness, imprecision, and publication bias. Based on the GRADE criteria, the evidence was categorized into four levels: high, moderate, low, and very low quality.

## Results

### Study selection

**Figure 1:**
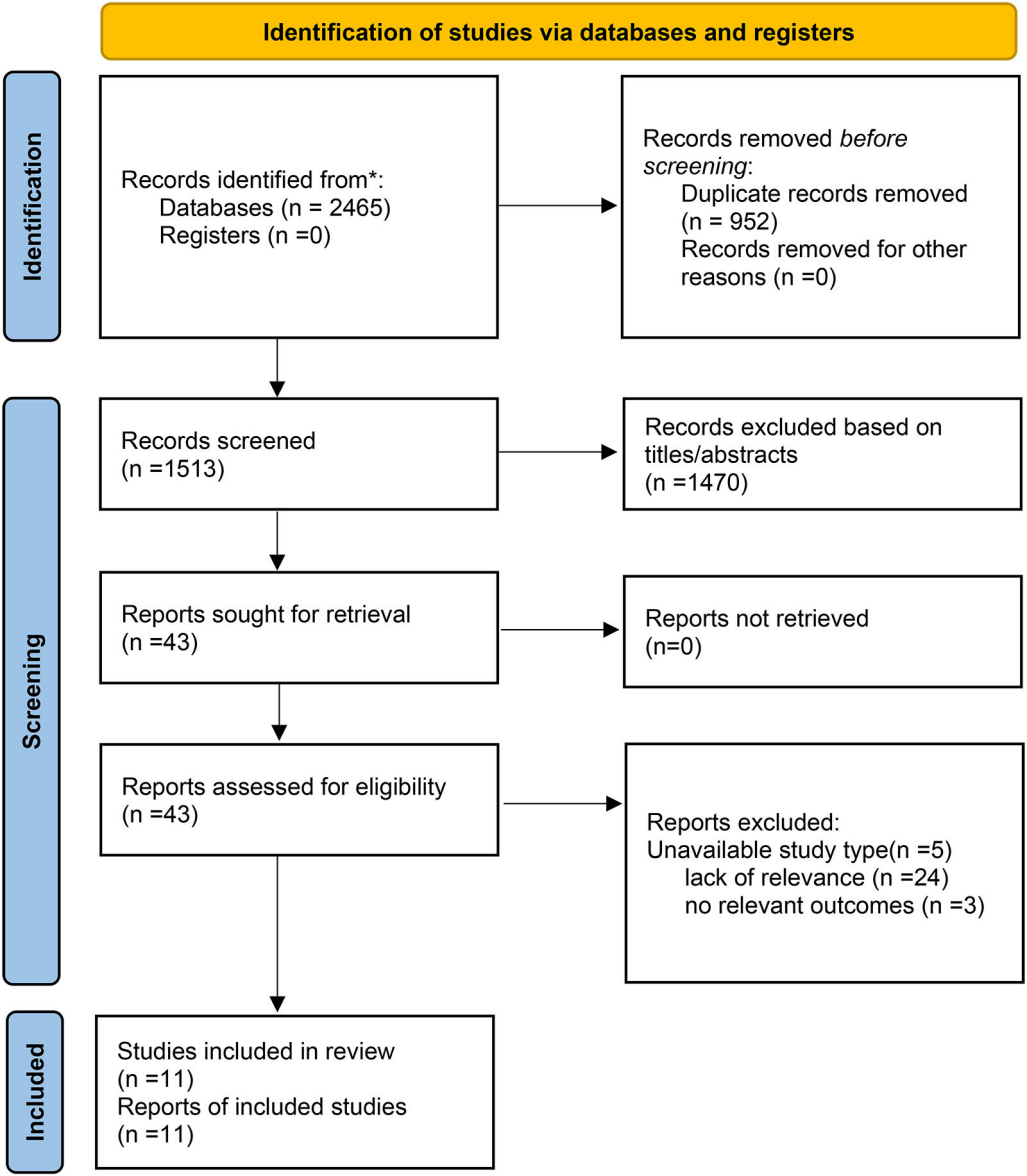
Search strategy.

In the initial screening, 2,465 citations were identified across the databases. After removing duplicates, 1,513 articles were evaluated based on their titles and abstracts. Subsequently, 43 articles were retrieved for full-text assessment. After a thorough examination, 5 articles were excluded due to unsuitable study types, 24 for lack of relevance, and 3 for not reporting relevant outcomes. Ultimately, 11 articles that met our eligibility criteria were included in the final analysis. A high level of concordance was observed among the reviewers in terms of data screening and integration (kappa = 0.81). Of the included studies, 4 were published in Chinese and 7 in English. The flow chart of the study selection is demonstrated in **Figure 1**.

### Characteristics of included studies

A total of 5650 patients were enrolled, with 3127 undergoing FET and 2523 undergoing fresh ET in the IVF/ICSI cycles. Six studies originated from China (15, 25–29), two from France (11, 12). and the remaining three from Canada, England, and Turkey, respectively (13, 14, 30). Of the eleven studies, two primarily addressed adenomyosis (12, 15), while the remaining nine focused on various types of EMS (8, 11, 13, 14, 25, 27–30). Among the EMS studies, three exclusively included patients with ovarian endometriomas, rASRM stage I-II EMS, or rASRM stage III-IV EMS, respectively (25, 26, 30). In four studies, the diagnosis of EMS was made via laparoscopy or laparotomy, while in five studies, it was determined using either laparoscopy or imaging examinations such as TVUS and magnetic resonance imaging. Adenomyosis was diagnosed by TVUS. Among the included studies, all involved clinical pregnancy rate, five involved embryo implantation rate (25, 26, 28–30), five involved ectopic pregnancy rate (25, 26, 28–30), ten involved live birth rate (11–13, 15, 25–30), and ten involved miscarriage rate (11, 13–15, 25–30). However, the definitions of miscarriage rate varied, with pregnancy loss ranging from 6 to 28 weeks, potentially introducing confounding factors. Therefore, we adopted the most common definition of miscarriage rate, specifically as a pregnancy loss occurring before 28 weeks after clinical pregnancy confirmation, and excluded four studies with differing definitions of miscarriage rate (13, 14, 26, 30). **Table 1** presents the characteristics of each study included and **Table 2** outlines the main results from each study included in the analysis.

**Table 1:** Baseline characteristics.

Study	Country	Study period	Study design	Type of disease	Intervention	Age (years)	BMI (kg/m^2^)	Infertility years	Diagnosis	EMS Phenotype or stage	Stimulation protocol	FET protocol	Oocyte retrieved	Outcomes
Tan2021 (14)	Canada	2014-2019	retrospective cohort study	EMS	FET(n=389)	35.9 ± 0.3	22.2 ± 0.1	2.8 ± 0.3	surgery	ASRM Stage I-IV	GnRH antagonist	HRT	7.4 ± 0.3	CPR, MR
					Fresh ET(n=339)	35.5 ± 0.2	22.4 ± 0.2	2.9 ± 0.3					8.2 ± 0.8	
Asoglu2020 (30)	Turkey	2014-2018	retrospective cohort study	EMS	FET(n=180)	33.1 ± 4.2	22.7 ± 2.4	2.9 ± 2.0	TVUS	Endometrioma	GnRH antagonist/long GnRH agonist	HRT	8.5 ± 3.5	IR, CPR, MR, LBR, EPR
					Fresh ET(n=135)	33.3 ± 3.4	22.9 ± 3.8	3.2 ± 2.0					8.5 ± 3.2	
Wu2019 (26)	China	2006-2017	retrospective cohort study	EMS	FET(n=506)	33.0 (30.0, 35.0)	20.7 (19.2, 22.4)	3.0 (2.0, 5.0)	Laparoscopy	ASRM Stage III-IV	NOS	NOS	7.0 (4.0, 12.0)	IR, CPR, MR, LBR, EPR
					Fresh ET(n=225)	32.0 (29.0, 35.0)	20.6 (19.1, 22.3)	4.0(2.0, 6.0)					8.0(5.0, 13.0)	
Mohamed2011 (13)	England	2000-2008	retrospective cohort study	EMS	FET(n=148)	NOS	NOS	NOS	Laparoscopy	NOS	long GnRH agonist	GnRH-a+HRT	NOS	CPR, MR, LBR
					Fresh ET(n=267)									
Bourdon2018 (11)	France	2012-2014	prospective cohort study	EMS	FET(n=135)	34.3 ± 4.1	22.8 ± 3.6	4.7 ± 2.7	TVUS/MRI/surgery	SUP, OMA, DIE	HRT	HRT	9.9 ± 7.0	CPR, MR, LBR
					Fresh ET(n=135)	34.3 ± 3.9	22.3 ± 3.3	4.4 ± 2.3					7.4 ± 4.3	
Wang2018 (25)	China	2010-2017	retrospective cohort study	EMS	FET(n=419)	30.4 ± 3.9	22.1 ± 2.3	4.0 ± 2.2	Laparoscopy	ASRM Stage I-II	long GnRH agonist	GnRH-a+HRT	15.1 ± 8.9	IR, CPR, MR, LBR, EPR
					Fresh ET(n=102)	31.2 ± 3.8	21.7 ± 1.9	4.1 ± 3.0					13.2 ± 8.0	
Yue2022 (27)	China	2014-2018	retrospective cohort study	EMS	FET(n=231)	31.3 ± 3.9	NOS	NOS	TVUS/Laparoscopy	NOS	GnRH antagonist/long or short GnRH agonist	NC/OI/HRT	13.2 ± 6.6	CPR, MR, LBR
					Fresh ET(n=231)	31.4 ± 3.9							8.3 ± 4.4	
Zhang2024 (29)	China	2011-2022	retrospective cohort study	EMS	FET(n=516)	31.6 ± 3.6	20.9 ± 2.5	4.5 ± 2.8	TVUS/Laparoscopy	NOS	NOS	NC/OI/HRT	NOS	IR, CPR, MR, LBR, EPR
					Fresh ET(n=645)	31.4 ± 3.5	21.0 ± 2.6	4.8 ± 2.6						
Dai2024 (28)	China	2017-2021	retrospective cohort study	EMS	FET(n=310)	31.8 ± 4.4	23.3 ± 3.1	4.9 ± 3.7	TVUS/surgery	ASRM Stage I-IV	ultralong/long GnRH agonist	GnRH-a+HRT/HRT	NOS	IR, CPR, MR, LBR, EPR
					Fresh ET(n=242)	30.5 ± 4.1	23.1 ± 3.3	4.7 ± 3.0						
Zhang2023 (15)	China	2018-2021	retrospective cohort study	AD	FET(n=98)	35.1 ± 4.7	22.9 ± 3.8	3.7 ± 2.3	TVUS	NOS	GnRH antagonist GnRH agonist/PPOS	NC/OI/HRT	5.0 (2.0, 10.0)	CPR, MR, LBR
					Fresh ET(n=91)	34.7 ± 3.9	23.8 ± 4.0	3.3 ± 2.1					7.0(4.0, 11.0)	
Bourdon2024 (12)	France	2018-2021	retrospective cohort study	AD	FET(n=195)	35.6 ± 3.4	23.9 ± 4.2	4.2 ± 2.7	TVUS	NOS	GnRH antagonist/long or short GnRH agonist	NC/OI/HRT	10.8 ± 6.7	CPR, LBR
					Fresh ET(n=111)	35.2 ± 3.5	23.5 ± 3.6	3.7 ± 2.5					7.3 ± 3.3	

ASRM, American Society of Reproductive Medicine; AD, adenomyosis; CPR, clinical pregnancy rate; DIE, deeply infiltrating endometriosis; EMS, endometriosis; FET, freeze-all embryo transfer; GnRH gonadotropin-releasing hormone; HRT, hormone replace treatment; IR, implantation rate; LBR, live birth rate. MR, miscarriage rate; MRI, magnetic resonance imaging; NC, natural cycle; NOS, not otherwise specified; OI, ovulation induction; OMA, ovarian endometrioma; PPOS, progestin-primed ovarian stimulation; SUP, superficial peritoneal endometriosis; TVUS, transvaginal ultrasound.

**Table 2:** Pregnancy outcomes of FET group compared with fresh group.

Study	Subjects (n)	Implantation rate (%)		Clinical pregnancy rate (%)		Miscarriage rate (%)		Ectopic pregnancy rate (%)		Live birth rate (%)	
		FET group	Fresh ET	FET group	Fresh ET	FET group	Fresh ET	FET group	Fresh ET	FET group	Fresh ET
Tan2021 (14)	728	NOS	NOS	40.9(159/389)	41.3(140/339)	NOS	NOS	NOS	NOS	NOS	NOS
Asoglu2020 (30)	315	64.4(116/180)	49.6(67/135)	63.3(114/180)	50.4(68/135)	NOS	NOS	0.9(1/114)	4.4(3/68)	56.1(101/180)	39.9(55/135)
Wu2019 (26)	731	34.4(332/964)	25.5(125/491)	51.8(262/506)	44.0(99/225)	NOS	NOS	0.3(1/262)	1.0(1/99)	45.3(229/506)	36.4(82/225)
Mohamed2011 (13)	415	NOS	NOS	18.2(27/148)	20.2(54/267)	NOS	NOS	NOS	NOS	16.9(25/148)	19.5(52/267)
Bourdon2018 (11)	270	NOS	NOS	37.0(50/135)	28.9(39/135)	16.0(8/50)	38.5(15/39)	NOS	NOS	28.9(39/135)	15.6(21/135)
Wang2018 (25)	521	26.2(289/1105)	28.8(64/222)	43.0(180/419)	47.1(48/102)	6.7(12/180)	14.6(7/48)	3.9(7/180)	4.2(2/48)	35.6(149/419)	28.4(29/102)
Yue2022 (27)	462	NOS	NOS	57.1(132/231)	43.7(101/231)	18.2(24/132)	13.9(14/101)	NOS	NOS	46.8(108/231)	37.7(87/231)
Zhang2024 (29)	1161	46.3(478/1032)	42,2(545/1290)	66.7(344/516)	60.0(387/645)	13.7(47/344)	10.9(42/387)	1.5(5/344)	2.8(11/387)	56.6(292/516)	51.8(334/645)
Dai2024 (28)	552	41.5(231/556)	36.9(167/453)	57.4(178/310)	52.9(128/242)	16.9(30/178)	13.3(17/128)	0.6(1/178)	0.8(1/128)	47.4(147/310)	(110/242)
Zhang2023 (15)	189	NOS	NOS	33.7(33/98)	46.2(42/91)	27.3(9/33)	26.2(11/42)	NOS	NOS	19.4(19/98)	27.5(25/91)
Bourdon2024 (12)	306	NOS	NOS	41.0(80/195)	33.3(37/111)	NOS	NOS	NOS	NOS	27.2(53/195)	20.7(23/111)

ET, embryo transfer; FET, freeze-all embryo transfer; NOS, not otherwise specified.

### Meta analysis

#### Clinical pregnancy rate

**Figure 2:**
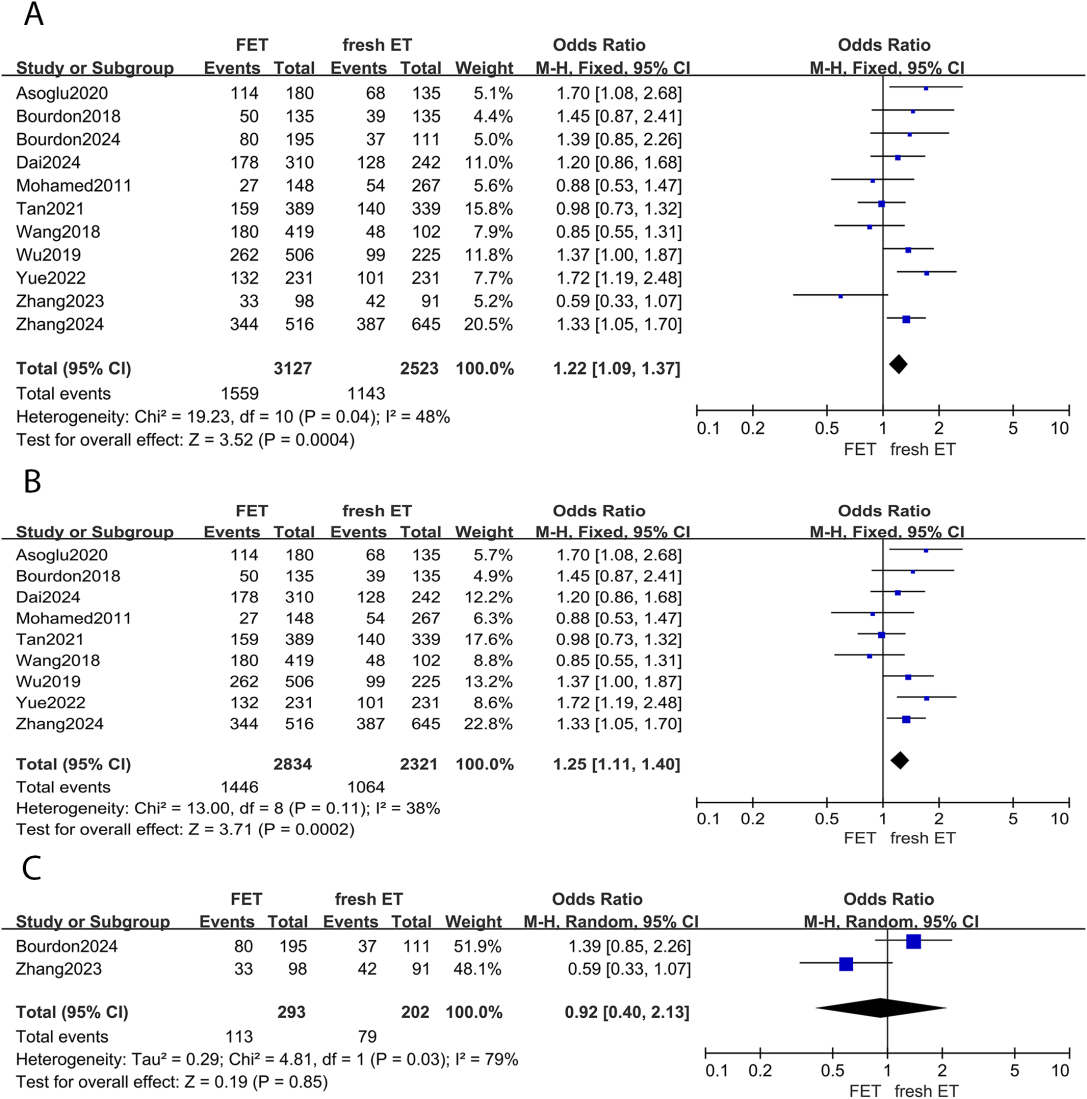
Forest plot for clinical pregnancy rate: **(A)** FET group versus fresh ET group in patients with endometriosis and adenomyosis; **(B)** FET group versus fresh ET group in patients with endometriosis; **(C)** FET group versus fresh ET group in patients with adenomyosis.

There were 11 studies investigating the difference in clinical pregnancy rate between FET and fresh ET groups. Compared to fresh ET, FET group improved clinical pregnancy rate (0R: 1.22; 95% CI: 1.09, 1.37; *p* <0.01; *I^2^* = 48%) (**Figure 2A**). In the subgroup analysis, the results of women with EMS were consistent with the overall analysis, observing a higher clinical pregnancy rate in FET group (0R: 1.25; 95% CI: 1.11, 1.40; *p* <0.01; *I^2^* = 38%) (**Figure 2B**). However, among women with adenomyosis, the clinical pregnancy rate did not significantly differ between the FET and fresh ET groups (0R: 0.92; 95% CI: 0.40, 2.13; *p* =0.85; *I^2^* = 79%) (**Figure 2C**).

#### Live birth rate

**Figure 3:**
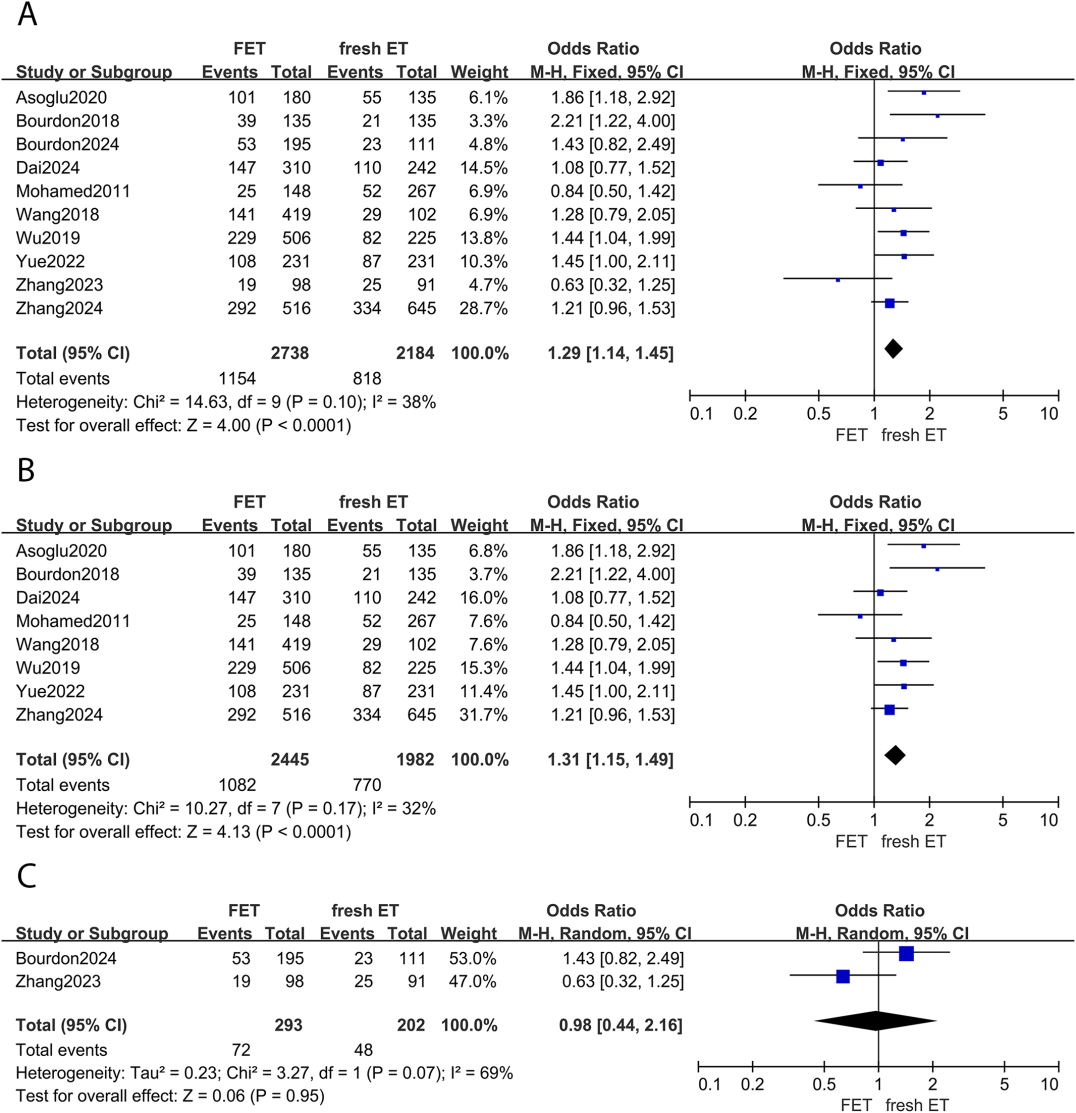
Forest plot for live birth rate: **(A)** FET group versus fresh ET group in patients with endometriosis and adenomyosis; **(B)** FET group versus fresh ET group in patients with endometriosis; **(C)** FET group versus fresh ET group in patients with adenomyosis.

Of the included studies, 10 reports investigated the live birth rate between different embryo transfer groups. A significant increase in live birth rates was observed in women undergoing FET cycles compared to those undergoing fresh ET cycles (OR: 1.29; 95% CI: 1.14, 1.45; p <0.01; *I^2^* = 38%) (**Figure 3A**). Subgroup analysis consistently showed a higher live birth rate in the FET group among women with EMS (OR: 1.31; 95% CI: 1.15, 1.49; *p* <0.01; *I^2^ =* 32%) (**Figure 3B**). However, for women with adenomyosis, the live birth rate was similar between groups (OR: 0.98; 95% CI:0.44, 2.16; p =0.95; *I^2^* = 69%) (**Figure 3C**).

#### Miscarriage rate

**Figure 4:**
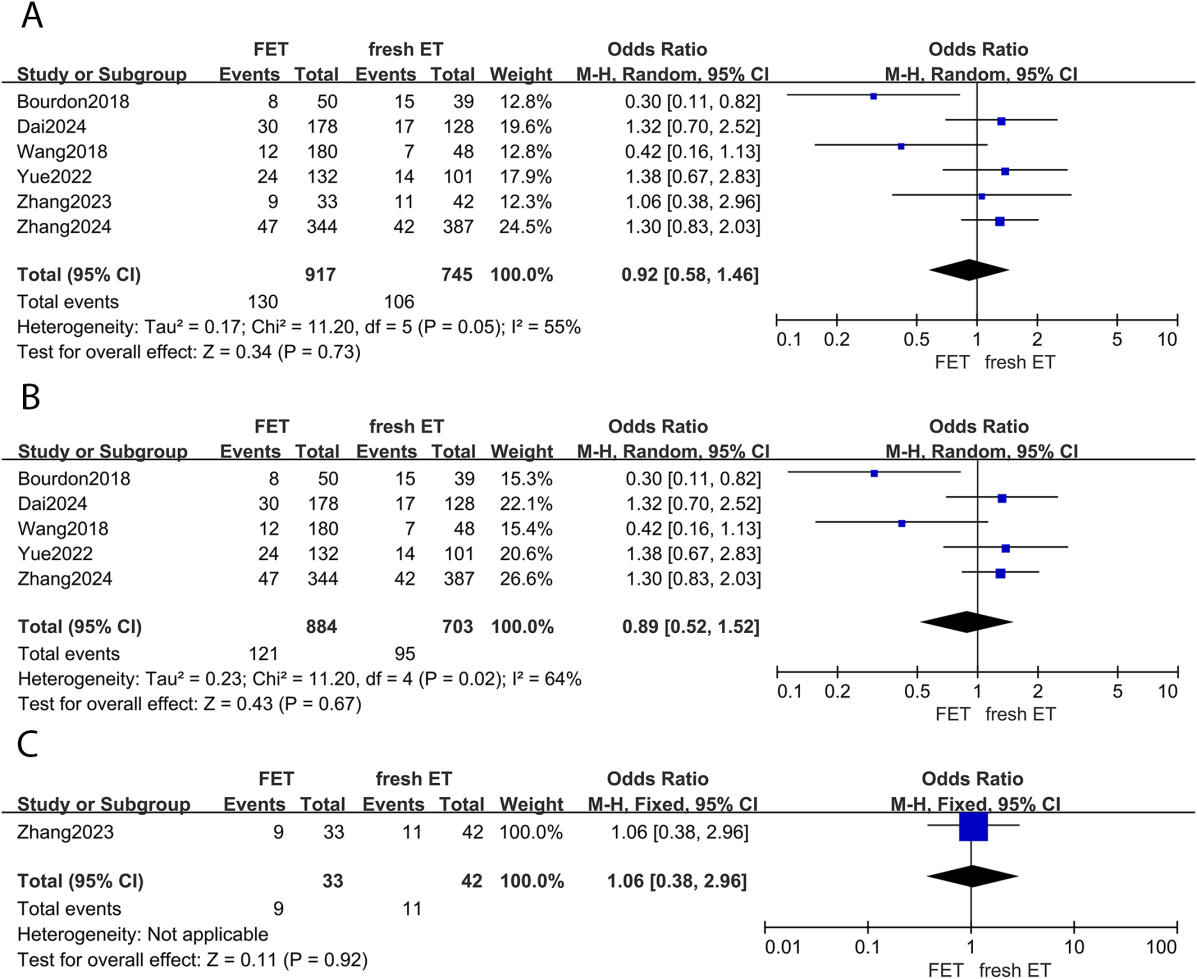
Forest plot for miscarriage rate: **(A)** FET group versus fresh ET group in patients with endometriosis and adenomyosis; **(B)** FET group versus fresh ET group in patients with endometriosis; **(C)** FET group versus fresh ET group in patients with adenomyosis.

Five studies provided data on miscarriage rates between the FET and fresh ET groups. The results indicated no statistical difference between groups (OR: 0.92; 95% CI: 0.58, 1.46; *p* =0.73; *I^2^* = 55%) (**Figure 4A**). This finding was consistent in the subgroup analysis focusing on patients with EMS (OR: 0.89; 95% CI: 0.52, 1.52; p =0.67; *I^2^* = 64%) (**Figure 4B**). Moreover, the sensitivity analysis of EMS did not alter the conclusion after eliminating any study. Similarly, among women with adenomyosis, the risk of miscarriage was comparable between the FET and fresh ET groups (OR: 1.06; 95% CI: 0.38, 2.96; p =0.92) (**Figure 4C**).

#### Implantation rate

Of the 11 studies included, 5 described the association between different embryo transfer types and implantation rates in patients with EMS. The combined analysis demonstrated that FET group exhibited a higher implantation rate compared to fresh ET group (OR: 1.27; 95% CI: 1.05, 1.54; *p* =0.01; *I^2^* = 62%) (**Supplementary Figure S2**). A sensitivity analysis was conducted to address heterogeneity, confirming the findings with consistent results after excluding the study by Wang et al. (25) (OR: 1.32; 95% CI: 1.18, 1.49; *p* <0.01; *I^2^* = 38%). Data regarding implantation rate specifically in women with adenomyosis were lacking.

#### Ectopic pregnancy rate

In this meta-analysis, 5 studies were included to investigate the ectopic pregnancy rate between FET and fresh ET groups in women with EMS. The finding indicated a similar ectopic pregnancy rate between groups (OR: 0.51; 95% CI:0.24, 1.07; *p* =0.08; *I^2^* = 0%) (**Supplementary Figure S3**). There were no available data to investigate the relationship between ectopic pregnancy rate and different embryo transfer types in women with adenomyosis.

### Publication bias assessment of the included studies

Funnel plots for each of the meta-analyses, as shown in **Supplementary Figure S1**, appeared to be symmetrical, except for the ectopic pregnancy rate, which suggested a potential, though minimal, publication bias likely due to the non-publication of smaller studies.

### Quality assessment of the included studies

The NOS used for assessing the quality of studies in this meta-analysis is presented in **Table 3**. The included studies received scores ranging from 7 to 9, indicating they were classified as high-quality studies with a low risk of bias.

**Table 3:** Quality assessment of cohort studies using the NOS.

Study	Selection				Comparability	Outcome	Follow-up long enough	Adequacy of follow up
	Representativeness of exposed cohort	Selection of non-exposed cohort	Ascertainment of exposure	Demonstration that outcome of interest was not present at start of study	Comparability of cohorts on the basis of the design or analysis	Assessment of outcome		
Tan2021 (14)	–	*	*	*	*	*	*	*
Asoglu2020 (30)	–	*	*	*	**	*	*	*
Wu2019 (26)	*	*	*	*	**	*	*	*
Mohamed2011 (13)	–	*	*	*	–	*	*	*
Bourdon2018 (11)	*	*	*	*	*	*	*	*
Wang2018 (25)	–	*	*	*	*	*	*	*
Yue2022 (27)	*	*	*	*	**	*	*	*
Zhang2024 (29)	*	*	*	*	*	*	*	*
Zhang2023 (15)	*	*	*	*	*	*	*	*
Bourdon2024 (12)	–	*	*	*	**	*	*	*
Dai2024 (28)	*	*	*	*	*	*	*	*

NOS, Newcastle–Ottawa Scale. * means one score, ** means two score.

### GRADE assessment

The quality of evidence for each outcome was evaluated using the GRADE approach as demonstrated in **Supplementary Figure S4**. The findings revealed that the clinical pregnancy and live birth rates, as well as the ectopic pregnancy rate, following different embryo transfer strategies for patients with EMS and adenomyosis, or EMS alone, were assessed as having moderate certainty of evidence. In contrast, all other outcomes were rated as low or very low certainty of evidence.

## Discussion

The objective of this study was to compare pregnancy outcomes between FET and fresh ET cycles in infertile patients with EMS and adenomyosis. The results of the meta-analysis involved 11 studies with a total of 5,650 patients, of which nine focused on endometriosis, while the remaining studies examined adenomyosis. In endometriosis-associated infertility patients undergoing different embryo transfer strategies, FET was associated with increased implantation, clinical pregnancy, and live birth rates, while no significant differences were observed in miscarriage and ectopic pregnancy rates. Compared to previous meta-analysis (31), our findings highlight that FET protocols improve clinical pregnancy rates while maintaining comparable miscarriage rates. These differences may be attributed to the inclusion of a greater number of studies in our analysis. In women with adenomyosis, IVF/ICSI outcomes were similar between FET and fresh ET groups. However, given that only two studies focused on pregnancy outcomes in adenomyosis, these findings should be interpreted with caution.

Due to the elusive nature of their pathogenesis, both EMS and adenomyosis are often referred to as “enigmatic diseases” (32). The question of whether EMS and adenomyosis represent two phenotypes of the same disease or are distinct entities remains a topic of ongoing debate (1, 32, 33). Dysfunction of the myometrial junctional zone and aberrations in the eutopic and heterotopic endometria suggest that adenomyosis and EMS may share a common origin (34). However, a shared origin does not imply they are identical, as they differ significantly in their histological, and clinical manifestations, as well as in their associated risk factors (32). Regardless of whether they are considered the same disease, it is clear that both conditions have a detrimental impact on women’s fertility.

EMS is associated with poor pregnancy outcomes during IVF/ICSI, the mechanisms are highly complex. In addition to alterations in pelvic anatomy and diminished ovarian reserve, changes in the immune microenvironment and reduced endometrial receptivity also play significant roles (35). Implantation is a pivotal aspect of assisted reproductive technology (ART). Supraphysiologic levels of estradiol and progesterone during COS could impair endometrial receptivity and lead to embryo-endometrium asynchrony, thereby reducing implantation rates during IVF/ICSI (36). Meanwhile, EMS, an estrogen-dependent disease, is characterized by inflammation, dysregulated differentiation of endometrial mesenchymal cells, and abnormal epigenetic marks in both the intracavitary endometrium and ectopic endometriotic tissue, all of which are associated with the imbalance between estrogen and progesterone (37). As a result, it is plausible to conjecture that the heightened steroid levels during COS and the intricate pathological characteristic of EMS itself might synergistically impede endometrial receptivity. The FET strategy, an alternative to fresh ET, allows for the separation of COS and embryo transfer. In this way, endometrial development can be controlled more precisely than in cycles of COS with gonadotropins (38). Additionally, embryos are transferred to an environment that has not been exposed to the supraphysiologic hormonal levels associated with COS. Consequently, it has been proposed that FET is advantageous for ART outcomes compared to fresh ET. Yue et al. included 462 patients with EMS to compare pregnancy outcomes between different embryo transfer methods. Their findings indicated that the cumulative clinical pregnancy rate was significantly higher in the FET group compared to the fresh ET group (27). Similarly, a meta-analysis involving six cohort studies revealed that in patients with endometriosis-related infertility, the live birth rate following FET was significantly higher than that of fresh ET. Additionally, the miscarriage rate was statistically lower in the FET group (31). Our study, which incorporated more recent research, confirmed part of these findings, demonstrating consistent improvements in pregnancy outcomes with the FET strategy in patients with endometriosis. However, the miscarriage rate in our analysis was comparable between the FET and fresh ET groups. We considered this discrepancy may arise from variations in the definition of miscarriage rate across studies. To minimize potential confounding, we only included studies that adhered to the most commonly accepted definition of miscarriage, excluding those with differing criteria. In addition, Tan et al. investigated early pregnancy outcomes in fresh versus freeze-all ET for patients with endometriosis, and the results showed comparable outcomes between the two groups (14). We believe that the observed differences may associated with the type of embryos transferred. The study by Tan et al. exclusively included patients who received blastocyst transfers. Previous research has shown that there are significant differences between blastocysts and cleavage-stage embryos in terms of embryo development and synchronization with the endometrium, which could potentially impact pregnancy outcomes (39–41). Additionally, variations in controlled ovarian stimulation protocols and differences in the staging and classification of endometriosis may also influence pregnancy outcomes (8).

In women with adenomyosis, infertility may arise from local endometrial inflammation due to alterations in the eutopic endometrium. Immunologic changes, fibrosis, local hyperestrogenism, and dysperistalsis of the myometrium could be responsible for altered endometrial receptivity and implantation (42, 43). ART is an effective method to improve pregnancy outcomes in infertile adenomyosis-associated patients. In this meta-analysis, we summarized the IVF/ICSI pregnancy outcomes after different embryo transfer strategies. The results indicated that the pregnancy outcomes were comparable between groups. A retrospective cohort analysis involving 306 infertile adenomyosis-associated patients compared the IVF/ICSI outcomes after FET or fresh ET strategies. It also revealed that the rate of clinical pregnancy, miscarriage, and live birth were not significantly different between the two groups (15). However, Bourdon et al. revealed that the FET groups showed higher cumulative ongoing pregnancy rate and cumulative live birth rate versus fresh ET groups in infertile patients affected by adenomyosis (12). They considered that a freeze-all strategy could be beneficial by avoiding the negative effects of ovarian stimulation on an already impaired endometrium. In addition, FET strategy offers an opportunity for gonadotropin-releasing hormone agonist (GnRH-a) pretreatment before embryo transfer, which potentially improve uterine cavity morphology and create a more favorable endometrial environment (44). Due to the limitations of the study design and the small sample size, more rigorous and large-scale multicenter randomized controlled trials are needed to explore this topic.

Overall, it is essential to follow women not only during their journey to achieve pregnancy but throughout their entire reproductive lifespan. Patients with adenomyosis and EMS may experience long-term reproductive health challenges that extend beyond conception, necessitating ongoing monitoring and management to optimize their overall gynecological and reproductive well-being.

### Strengths and limitations

This review has several limitations and strengths that may have influenced the findings. The strengths of our study include the following: it is a large-scale meta-analysis based on 11 studies with a novel focus on patients with EMS and adenomyosis. We conducted a systematic literature search adhering to strict inclusion and exclusion criteria, ensuring a rigorous methodology. In addition to reporting the pregnancy outcomes of clinical pregnancy and live birth, we also addressed key reproductive outcomes, such as ectopic pregnancy, which is particularly significant in the context of assisted reproductive treatments. Despite the precautions taken, our study is subject to certain limitations. The quality of each of the included studies varies. Since there are some significant differences in baseline characteristics concerning the age of patients, type of infertility, ovarian reserve function and ovarian protocols, among others. it could introduce confounding factors into the results. Additionally, the included studies differed in their ascertainment of EMS and adenomyosis, without explicitly distinguishing between the two conditions. This lack of distinction may have led to the analysis of gestational outcomes in a mixed group. Furthermore, our study did not examine pregnancy complications or obstetric outcomes, which warrant further investigation.

In clinical practice, factors such as uterine environment, endometrial receptivity, and the patient’s overall health should be carefully considered during IVF/ICSI cycles before embryo transfer. Based on current evidence, for women with EMS undergoing IVF/ICSI, clinicians might prioritize FET protocols over fresh embryo transfer. despite the potential for increased financial costs. Stimulation protocols should be adjusted to optimize oocyte retrieval and subsequent cryopreservation for FET cycles. For women with adenomyosis, pregnancy outcomes appear comparable between FET and fresh ET strategies. Clinicians should evaluate uterine abnormalities using imaging or hysteroscopy to guide decisions regarding endometrial preparation and the appropriate type of embryo transfer.

Heterogeneity exists across the studies due to substantial variations in study design, participant characteristics, and sample size. Although subgroup analyses for EMS and adenomyosis were performed to mitigate some of the heterogeneity, differences in the types of observational studies and patient characteristics, particularly variations in disease severity among EMS or adenomyosis patients, contribute to variability in baseline data, potentially impacting the outcomes. Consequently, randomized controlled trials focusing on different subtypes and severity levels of EMS and adenomyosis are warranted to better evaluate the efficacy and applicability of various embryo transfer strategies.

## Conclusion

In IVF/ICSI, the FET strategy has been associated with more favorable reproductive outcomes compared to the fresh ET strategy in women with EMS, whereas in women with adenomyosis, pregnancy outcomes were comparable between the FET and fresh ET groups. However, the current evidence remains limited. More investigations are underscored to confirm our findings.

## Data Availability

The original contributions presented in the study are included in the article/**Supplementary Material**. Further inquiries can be directed to the corresponding author/s.

## Supplementary material

Supplementary Figure 1 Funnel plot.

Supplementary Figure 2 Forest plot for implantation rate in the FET group versus fresh ET group in patients with endometriosis.

Supplementary Figure 3 Forest plot for ectopic pregnancy rate in the FET group versus fresh ET group in patients with endometriosis.

Supplementary Figure 4 Summary of findings (GRADE).
