# Supplementary figures and images for: Pregnancy outcomes in freeze-all versus fresh embryo transfer cycles of women with adenomyosis and endometriosis: a systemic review and meta-analysis
Source: Front Endocrinol (Lausanne). 2025 May 14;16:1507252. doi: 10.3389/fendo.2025.1507252 (PMC12116353; doi:10.3389/fendo.2025.1507252)

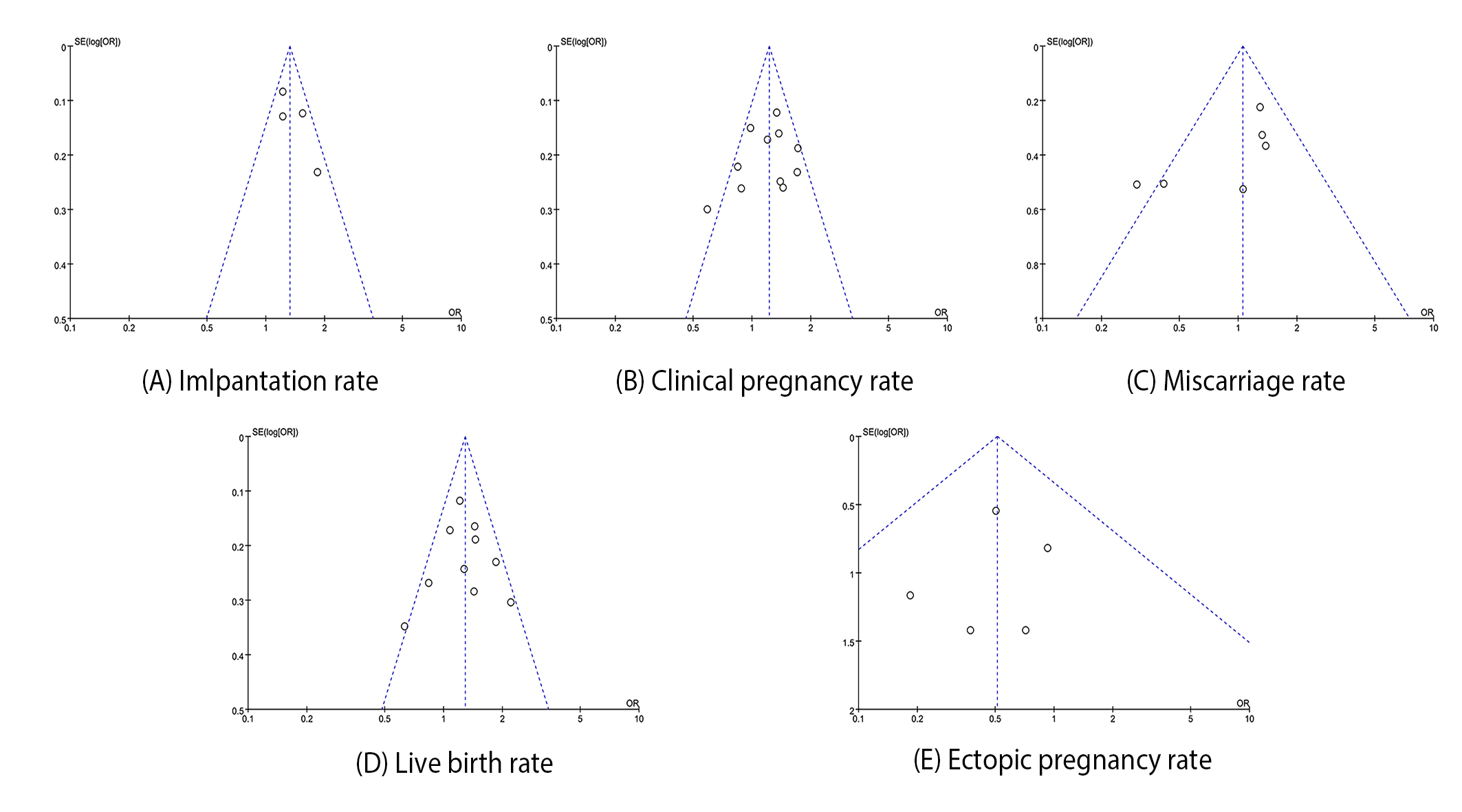

Supplement: Supplementary Figure 1 — Funnel plot. [file Image1.tif]

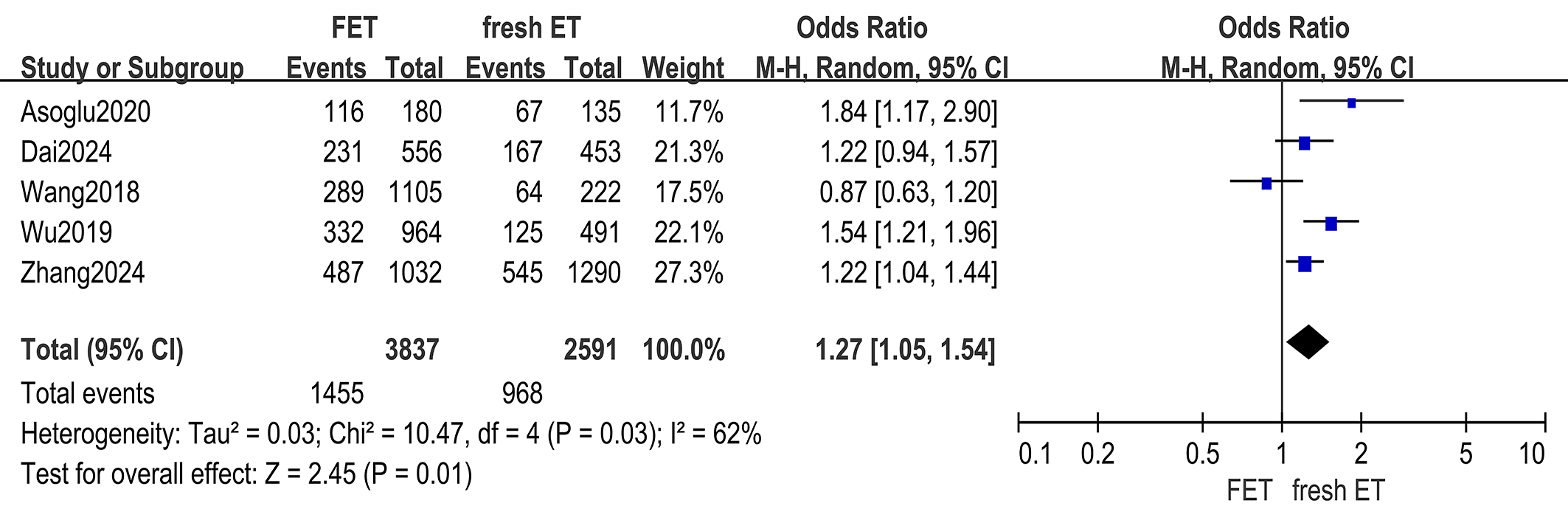

Supplement: Supplementary Figure 2 — Forest plot for implantation rate in the FET group versus fresh ET group in patients with endometriosis. [file Image2.tif]

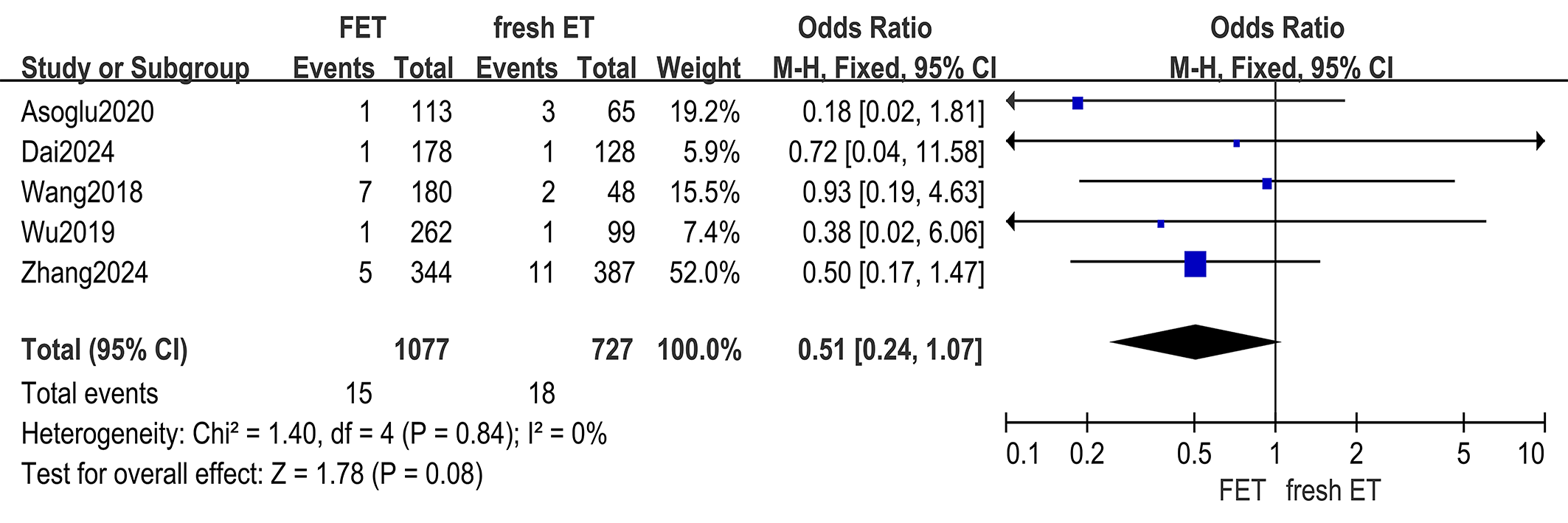

Supplement: Supplementary Figure 3 — Forest plot for ectopic pregnancy rate in the FET group versus fresh ET group in patients with endometriosis. [file Image3.tif]

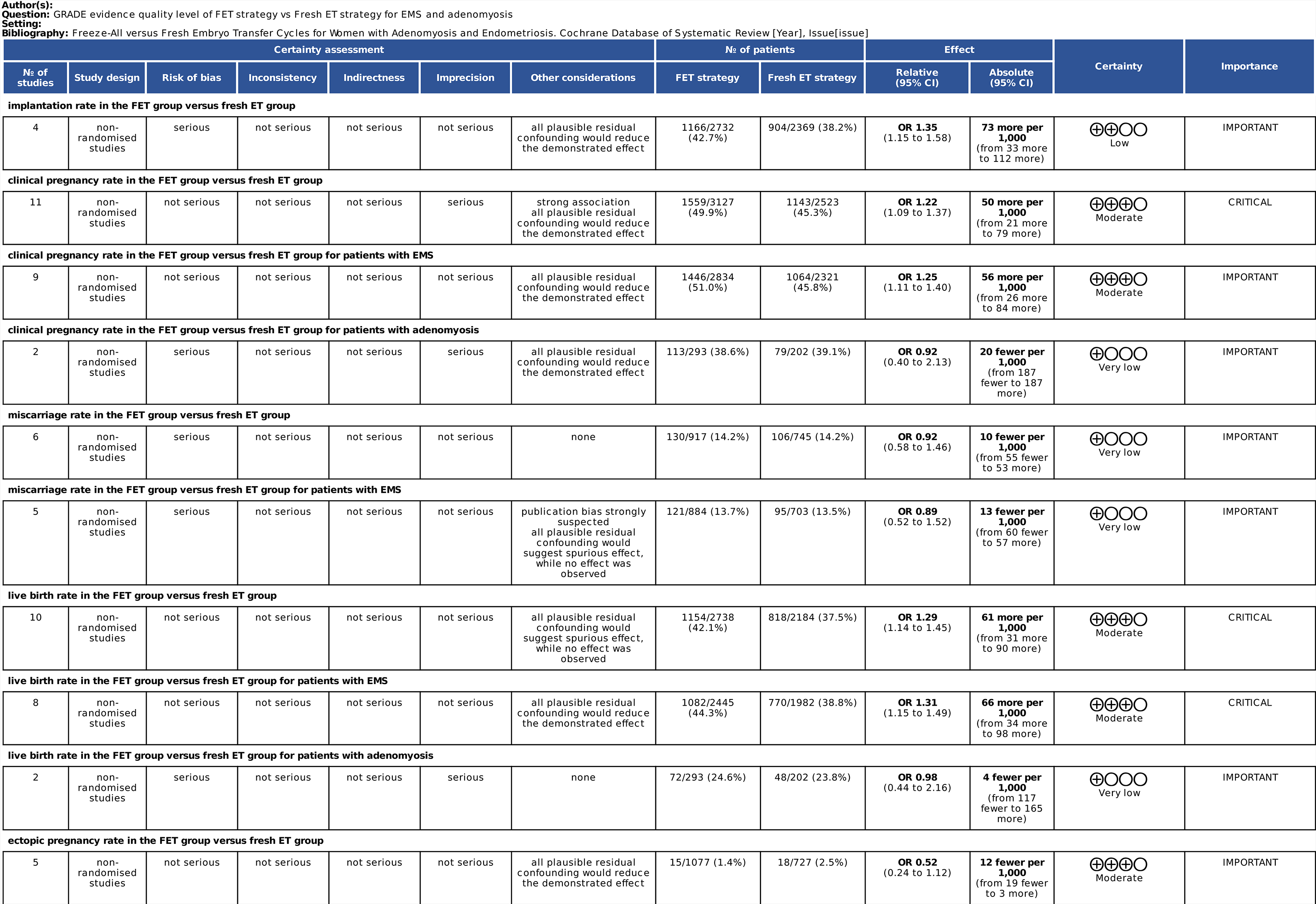

Supplement: Supplementary Figure 4 — Summary of findings (GRADE). [file Image4.tif]
